# Simulation and Optimization of Surface Roughness and Process Performance during Machining of HSS by Micro-WEDM Technology

**DOI:** 10.3390/mi15030372

**Published:** 2024-03-09

**Authors:** Ľuboslav Straka, Ivan Čorný

**Affiliations:** 1 Department of Automobile and Manufacturing Technologies, The Technical University of Kosice, Sturova 31, 080 01 Presov, Slovakia; 2 Department of Process Engineering, The Technical University of Kosice, Sturova 31, 080 01 Presov, Slovakia; ivan.corny@tuke.sk

**Keywords:** main technological parameters (MTP), optimization, performance, simulation, surface roughness, micro-wire electrical discharge machining (micro-WEDM)

## Abstract

When machining high-speed steels (HSS) with micro-wire electrical discharge machining (micro-WEDM), high surface quality is achieved as standard. The value of the roughness parameter Ra is less than 0.2 μm. However, the problem is the performance of the electroerosion process (MRR), which is low. This problem is related to the mechanical and physical properties of the HSS in combination with the setting of the main technological parameters (MTP). The proposed solution to eliminate this problem relies on the selection of proper procedures for the determination of optimization criteria in relation to Ra and MTP, with the inclusion of properties of the machined material. The solution consisted in the identification of four significant physical (*ρ*, *κ*) and mechanical (Rm, HRC) indicators of HSS properties, on the basis of which a suitable combination of the process output parameters Ra and MRR can be determined through established mathematical regression models using simulation and optimization. In the next step, the proper values of the MTP output process parameter settings, which correspond to the optimized output parameters Ra and MRR during machining of HSS by micro-WEDM technology, were then obtained by the same approach.

## 1. Introduction

Micro-wire electrical discharge machining (micro-WEDM) technology is used to cut complex three-dimensional profiles in metallic materials with conductivity higher than 0.01 Siemens·cm^−1^ [1,2]. It is also used in cases where it is necessary to machine complex contours with high quality of the machined surface in terms of roughness parameters and at the same time with high dimensional accuracy [3,4,5,6,7]. Its principle is based on a controlled electric discharge that occurs between two electrodes (cathode and anode) in the presence of a suitable dielectric environment. The cathode is usually represented by the machined material and the anode by the tool electrode. An electric discharge between the cathode and the anode always occurs when the basic conditions are met [8]. With continuous electric discharges, a temperature of 8000 to 12,000 °C is reached in the spark gap, thanks to which the metal particles from the machined material melt or evaporate, and the residues are subsequently washed away with a dielectric liquid [9,10]. The wire tool electrode is also important in the micro-WEDM process because it affects the cutting speed, dimensional accuracy, and the quality of the machined surface [11]. Micro-WEDM uses wire electrodes with a diameter of 0.05–0.1 mm made of Ms, Cu, Fe, Mo, W, and other materials [12].

It is general knowledge that the surfaces machined by micro-WEDM technology are characterized by good quality, but the overall performance of the electrical discharge process when machining is very low. However, this is contrary to the current trend, which is oriented towards achieving high quality of the machined surface and at the same time high performance of the process [13,14,15,16,17,18,19,20,21]. The low performance of the electrical discharge process lies in the physical nature of the material removal method and, at the same time, in the current approach and method of controlling the electrical discharges that take place between the tool electrode and the processed material. One of the suitable approaches for solving the given problem can be the search for a unique combination of levels of input factors that can in all circumstances have a favorable impact on important output quality indicators of the machined surface while maintaining high performance of the electrical discharge process [22,23,24,25]. The roughness of the machined surface is coupled with its texture and integrity, and at the same time defines the final geometry of the workpiece. Therefore, it is desirable that the roughness of the machined surface be as low as possible [26,27,28,29,30,31]. Kiyak, in his experimental research [32], observed that increasing of the pulse on-time duration, voltage of discharge, and feed rate of the wire electrode contribute to the increase of the crater diameter and crater depth, thereby increasing the roughness of the eroded surface. In contrast, by reducing them, he observed a substantial improvement of the machined surface roughness during micro-WEDM.

In addition to the roughness of the machined surface in WEDM, an important output indicator is also the material removal rate (MRR) which, according to Tosun et al. [33], is affected mainly by the peak current *I*, the voltage of discharge *U*, and the pulse on-time duration *t_on_*. Several authors [34,35,36,37,38,39], based on the results of their experimental research, also claim that the material removal rate (MRR), dimensional accuracy (DA), and surface roughness (SR) are important output indicators that define the quality of the machined surface and the performance of the WEDM process. At the same time, they claim that these output indicators of the electrical discharge process can be changed by varying the input technological parameters, such as the peak current *I*, voltage *U*, flushing pressure of the dielectric liquid *p*, the pulse on-time duration *t_on_*, the pulse off-time duration *t_off_*, the properties of the machined material and wire electrode, wire electrode tension, wire feed rate, and more. The mutual combination of the mentioned input technological parameters then generates craters in the machined material, which define the resulting quality of the machined surface as well as the performance of the electrical discharge process. Kozák et al. [40] found that the electrical resistance between the workpiece and the wire electrode changes during machining of low-conductivity material, as a result of which the material removal rate and the roughness of the machined surface are affected. Another approach to solving the problem associated with the low productivity of the electrical discharge process is its optimization. This was partly attempted by Pradhan et al. [41], who in their experiment applied the response method of selected input parameters on the quality of the machined surface. They found out which parameters are involved in maximizing the performance of the electrical discharge process. In this direction, the research was extended by Meena et al. [42], who also performed a mutual optimization of the performance indicators of the electrical discharge process depending on the selected input technological and process parameters. In addition to these researchers, many others have attempted to investigate the effects of various input factors and their levels on the output performance parameters of the electrical discharge process. However, their research was often limited to selected types of materials in the context of machining performance parameters with subsequent modeling of the material properties of the workpiece and the tool electrode [43,44,45,46,47,48,49,50]. In their work, Mahapatra et al. [51] evaluated relations and response control factors in WEDM such as MRR and cutting gap width by the Taguchi method. They identified the peak current, pulse on-time and off-time duration, pulse frequency, wire electrode feed rate, wire tension, and dielectric flow as important input parameters of the machining process that affect performance parameters. For these parameters, they used a genetic algorithm to optimize the wire electrode machining process with multiple responses.

Various complex mathematical models and simulation methods have been created to model WEDM processes [52,53,54,55]. Babu et al. [56] developed an empirical model using various process parameters to simulate the roughness of the machined surface. The results of the experiments demonstrated that the roughness of the machined surface increases with increasing *t_on_* and decreases with increasing *t_off_*. Phate et al. [57] analyzed the effect of input variable parameters *t_on_*, *t_off_*, *I*, and *WF* in WEDM Al/SiCp on MRR and Ra using artificial neural networks (ANN) and dimensional analysis techniques. They found out that higher thermal conductivity and *t_on_* contribute to their improvement. They also found that *t_on_* has the largest effect on MRR and Ra, followed by *I* and *WF*.

Many researchers have dealt with dimensional accuracy, machined surface roughness Ra, MRR, as well as designing models to predict high dimensional accuracy, low machined surface roughness, and high performance with optimal input parameters in WEDM. However, a research gap has arisen in the area of optimization of the output-dependent quality indicator of the machined area and the performance indicator of the electrical discharge process in WEDM of high-speed steels (HSS) depending on the MTP and the properties of the machined material. These reasons led us to perform experimental research, the goal of which was to achieve significant progress in optimizing the quality of the machined surface while maintaining high productivity of the electrical discharge process. At the same time, we would like to contribute to the database of already existing knowledge through clear formulations of particular laws in relation to the processes that take place directly on the machined surface during the electrical discharge [58,59,60,61,62,63,64,65].

Based on the analysis of the current state of the art in the field of optimization of the output quality indicators of the machined area and the output performance parameters of the electroerosion process in micro-WEDM, a research gap has been identified. This lies in the absence of effective approaches to the determination of optimization criteria in relation to Ra and MTP in conjunction with the properties of the machined material. Therefore, the proposed solution consisted in the identification of significant input variable parameters related to the physical and mechanical parameters of the HSS. On the basis of their identification, mathematical regression models are subsequently proposed, through which the proper combination of process output parameters Ra and MRR can be determined using simulation and optimization. The optimization of the output quality indicator of the machined surface Ra primarily respects the maximization of the performance parameter MRR during machining of HSS with micro-WEDM technology. Analogously, in the next step, the proper values of the settings of the significant MTP process output parameters are subsequently obtained, which correspond to the optimized output parameters Ra and MRR. The experiment was designed emphasizing an orderly approach to obtain the optimal output-dependent process parameters in micro-WEDM and appropriate values for the settings of the significant MTPs. The simulation and prediction of input-independent variables as well as output-dependent variables in machining of HSS by micro-WEDM was carried out by the means of the proposed regression mathematical models, with respect to achieving favorable machined surface roughness Ra and high performance of the electroerosion process.

## 2. Materials and Methods

### 2.1. Design and Conditions of the Experiment

The experimental samples were made from high-speed steels marked EN HS 3-2-2 (W.-Nr. 1.3333), EN HS 10-5-3-10 (W.-Nr. 1.3207), and EN HS6-5-2C (W.-No. 1.3343). These are high-alloy steels with a high content of Cr, Mo, V, and W alloying elements. The material of the experimental samples was heat-treated before micro-WEDM by quenching according to the relevant quenching diagrams with an orientation on achieving the maximum hardness of the base material, followed by tempering to remove internal stresses.

On the basis of the literature review mentioned at the introduction of the paper, it can be concluded that the quality of the machined surface in terms of the roughness parameter Ra and the productivity in terms of the MRR parameter in micro-WEDM, in addition to the main technological parameters (MTP), are also influenced by the physical and mechanical properties of the machined material. Regarding the physical properties, these parameters are mainly the specific electric resistance ρ and thermal conductivity κ. Regarding the mechanical properties, these parameters are mainly the tensile strength Rm and achievable hardness after refining HRC. Table 1 shows the basic mechanical and physical properties of the HSS that were used in the production of the experimental samples.

From the overview given in Table 1, it can be observed that the highest value of the parameter ρ = 0.80 Ω·mm^2^·m^−1^ was in the high-speed steel EN HS 10-5-3-10; in contrast, the lowest value ρ = 0.54 Ω·mm^2^·m^−1^ was in the high-speed steel EN HS6-5-2C. The highest value of the parameter κ = 21 W·m^−1^·K^−1^ at temperature of 20 °C was in the high-speed steel EN HS6-5-2C; in contrast, the lowest value κ = 19 W·m^−1^·K^−1^ at temperature of 20 °C was in the high-speed steel EN HS 10-5-3-10. High-speed steel EN HS6-5-2C had the highest value of the parameter Rm = 1158 MPa; on the other hand, high-speed steel EN HS3-3-2 had the lowest value of Rm = 757 MPa. High-speed steel EN HS 10-5-3-10 had the highest achieved hardness of 67HRC after heat treatment; on the other hand, the lowest hardness value of 62HRC after heat treatment was achieved by high-speed steel EN HS6-5-2C.

The values of the physical and mechanical properties of the given HSS were substantially influenced by their chemical composition. The following Table 2 shows the basic chemical composition of the HSS that were used in the experiment to make the experimental samples.

From the overview given in Table 2 it can be observed that the highest value of wt % of element C was in the high-speed steel EN HS 10-5-3-10; in contrast, the lowest value was in the high-speed steel EN HS6-5-2C. This confirms the claim regarding the dependence of selected physical and mechanical properties of HSS on their chemical composition.

As mentioned in the introduction, the quality of the machined surface in terms of the roughness parameter Ra and the productivity of MRR of the electrical discharge process depend on the combination of MTP settings, in addition to the physical and mechanical properties of the machined material and its chemical composition. Based on the analysis of the literature review mentioned in the introduction of the article, it can be concluded that the peak current *I*, the pulse on-time duration *t_on_*, the associated pulse off-time duration *t_off_*, and the voltage of discharge *U* had significant impact on the output indicators of the electrical discharge process. The following Table 3 provides an overview of the basic levels of MTP settings according to DoE 4-factor analysis at three levels of settings of the input dependent parameters of the process; namely, the peak current *I*, the pulse on-time duration *t_on_*, the pulse off-time duration *t_off_*, and the voltage of discharge *U*, which in total represent 27 experimental samples made of high-speed steels EN HS 3-2-2, EN HS 10-5-3-10, and EN HS6-5-2C using micro-WEDM technology.

From the overview in Table 3, it is evident that the expected value of the output quality parameter of the machined surface given by the surface roughness parameter Ra in micro-WEDM of HSS reflected the expected values of the output performance parameter of the electrical discharge process MRR. When setting MTP peak current *I* and pulse on-time duration *t_on_* on the upper level and parameters’ pulse off-time duration *t_off_* and voltage of discharge *U* on the lower level, a high value of the output power parameter MRR of the electrical discharge process was expected, and at the same time a high value of the output parameter of the roughness of the machined surface Ra. When setting MTP peak current *I* and pulse on-time duration *t_on_* on the lower level and parameters’ pulse off-time duration *t_off_* and voltage of discharge *U* on the upper level, a low value for the output power parameter MRR of the electrical discharge process was expected and at the same time a low value of the output parameter of the roughness of the machined surface Ra.

**Figure 1:**
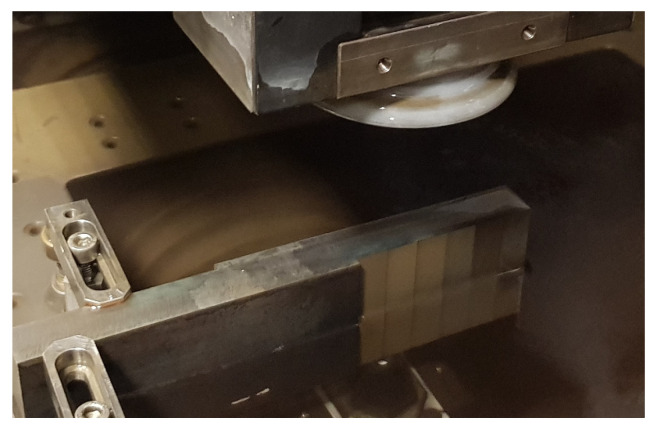
Production of experimental samples.

The experimental samples were made (Figure 1) on an electrical discharge machine CHMER EDM G32F (CHMER Corp., Taichung City, Taiwan). It was an autonomous electrical discharge device. The following Table 4 lists the basic technical parameters of the used electrical discharge machine.

The production of experimental samples from HSS was carried out on the CHMER EDM G32F electrical discharge machine in the presence of a dielectric liquid based on deionized water with an electrical conductivity of less than 10 μS·cm^−1^.

**Figure 2:**
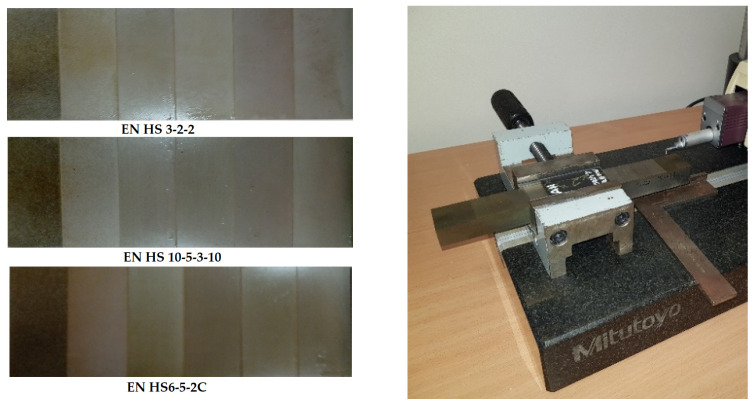
Measurement of the roughness parameter Ra of the eroded surface of the samples using the Mitutoyo Surftest SJ 400 measuring device.

The measurement of the output-dependent quality parameter of the roughness of the machined surface Ra after micro-WEDM on experimental samples from HSS (Figure 2) was carried out using the Mitutoyo Surftest measuring device SJ 400 (Mitutoyo, Kawasaki, Japan).

In order to eliminate the influence on the measurement of the surface roughness parameter Ra due to changes in the surface microstructure described by Pramanik et al. [66], the eroded surface of the experimental HSS samples was adjusted by additional treatment. The treatment process consisted of immersing the samples in 90% H_3_PO_4_-based solution with 10% mordant at a constant temperature of 20 °C. At the same time, the deposit from the wire electrode was removed from the surface of the experimental samples. This layer was removed from the eroded surface by spraying the solution based on water and ammonia at a concentration of 0.9 g·cm^−3^ in a ratio of water:ammonia = 9:1 with the addition of ammonium persulfate and sodium phosphate. Residual contaminants were removed from the surface by blasting with glass beads with a diameter of 50 μm.

### 2.2. Statistical Analysis of Measured Values

As part of the evaluation of the experimentally recorded values of output-dependent variables related to the quality of the machined surface in terms of the Ra parameter and the performance of the electrical discharge process in terms of the MRR parameter in micro-WEDM of HSS, a series of sequential steps was implemented. The reason was the fact that the results of experimentally recorded values are usually characterized by a highly asymmetric distribution and unconventional dispersion [67,68,69]. Therefore, in the first step, an exploratory data analysis was applied, which excluded anomalies in the obtained results of experimental measurements. It was mainly about specifics in the form of data distribution, exclusion of the occurrence of outliers, or revealing the local concentration of measured data. In the next step, the requirements for the set of measured data were verified due to the application of three different types of HSS. Finally, through confirmatory analysis, the verification of the measured data was carried out with the application of parameter estimation. The sampling analysis procedure was aimed at determining the objective mean value of a representative selection from the results of experimental measurements of the output performance parameter of the productivity of the electrical discharge process MRR and the qualitative parameter of the machined area Ra in micro-WEDM of HSS. The results of individual experimental measurements of the mentioned parameters were evaluated using standard statistical methods (Shapiro–Wilk test), the aim of which was to examine the normality of the data set and subsequently to identify outliers and extreme values (Grubs and Dixon test). This analysis was applied to the results of all the recorded data, both for the recorded values of the parameters of the roughness of the machined surface Ra, as well as for the recorded values of the performance parameter of the electrical discharge process MRR. In the case of the recorded data where the presence of outliers or extreme values was confirmed, based on the analysis, and it was not possible to establish a normal distribution, even in cases where the normality of the distribution of the data was not proven even after the exclusion of the confirmed outliers, exponential and Box–Cox transformations were performed, which ensured the correctness of the statistical analysis of experimentally measured values of monitored output-dependent parameters Ra and MRR in micro-WEDM of HSS [70,71,72,73,74].

**Figure 3:**
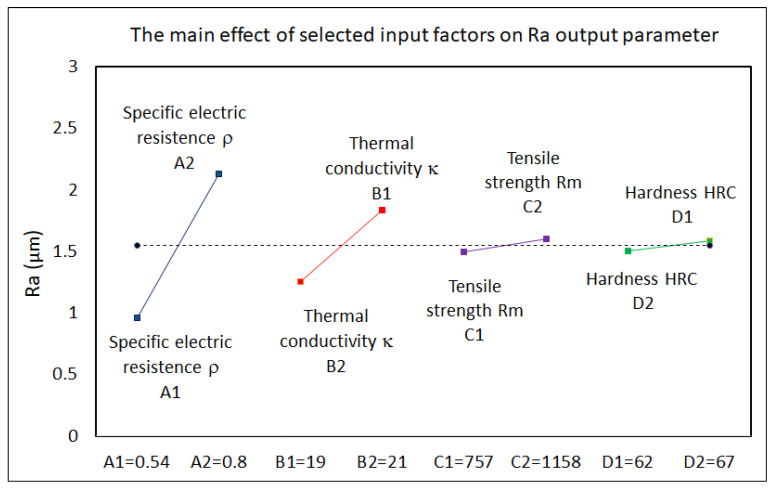
Analysis of the influence of selected input factors (physical and mechanical properties of HSS) on the Ra parameter in micro-WEDM.

**Figure 4:**
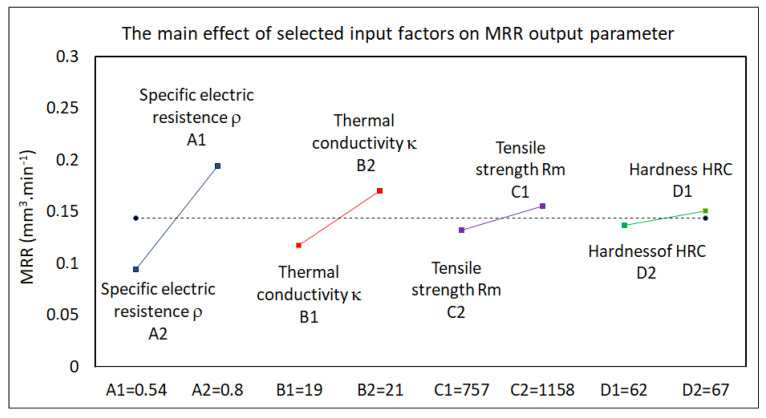
Analysis of the influence of selected input factors (physical and mechanical properties of HSS) on the MRR parameter in micro-WEDM.

Based on the analysis of the experimentally recorded data, it was found that the output quality parameter Ra and performance parameter MRR were dependent on many input factors in the electrical discharge process. They can be divided into two groups. The first group consisted of parameters related to the physical and mechanical properties of the machined material. The second group consisted of MTP variables. Their mutual combination then defined the resulting value of the mentioned output-dependent parameters of the electrical discharge process. At the same time, each of these input parameters participated with a certain share in the value of the output-dependent parameter. The results of the performed factor analysis of the influence of selected input variable factors (physical and mechanical properties of the machined material) on the output-dependent parameter Ra of the machined surface and the MRR parameter of the electrical discharge process in micro-WEDM of HSS are shown in the graphs in Figure 3 and Figure 4.

The following facts were identified from the analysis of the influence of selected input factors (physical and mechanical properties of HSS) on the parameters Ra and MRR in micro-WEDM. It was found that the thermal conductivity and specific electrical resistance of the material had the greatest influence on the output-dependent parameter Ra of the machined area and the performance parameter of the electrical discharge process when machining HSS with micro-WEDM technology. In contrast, the hardness of the machined material had the least influence. Therefore, only the parameters’ thermal conductivity *κ* and specific electric resistance *ρ* were taken into account during the design of mathematical regression models.

**Figure 5:**
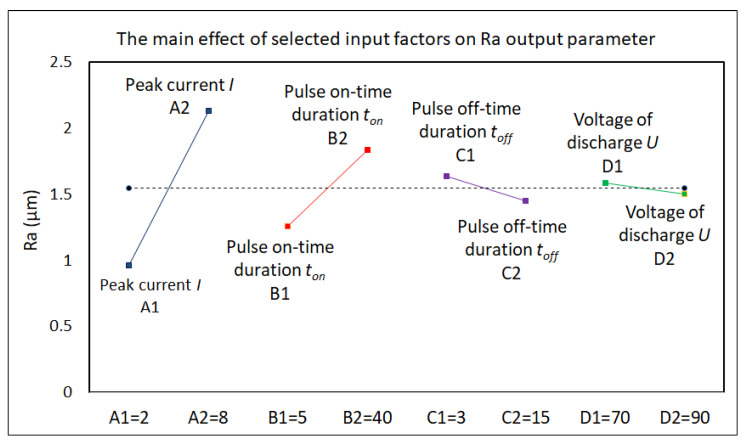
Analysis of the influence of selected MTP input factors on the parameter Ra in micro-WEDM of HSS.

**Figure 6:**
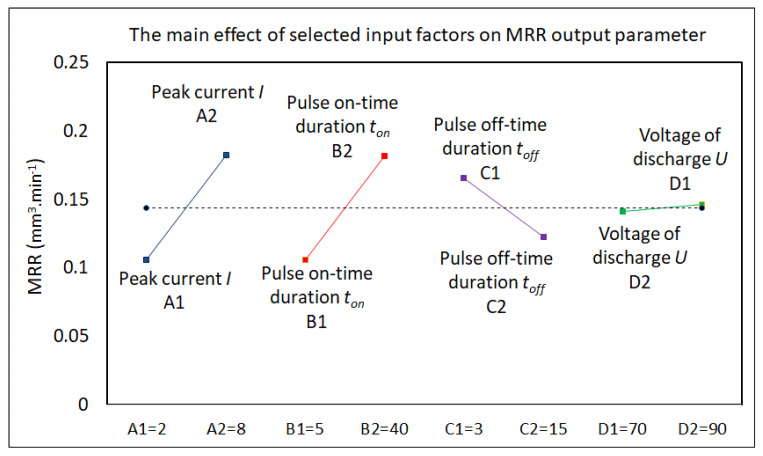
Analysis of the influence of selected MTP input factors on the parameter MRR in micro-WEDM of HSS.

Subsequently, a factor analysis of the influence of selected MTP input factors on the quality of the machined surface and the productivity of the electrical discharge process in the machining of high-speed steels with the micro-WEDM technology was performed. The results of the performed factor analysis of the influence of selected input variables of the MTP factors on the output-dependent parameter Ra of the machined surface and the parameter MRR of the electrical discharge process in micro-WEDM of HSS are shown in the graphs in Figure 5 and Figure 6.

The following facts were identified from the factor analysis of the influence of selected MTP input variables on the output-dependent parameters Ra and MRR in micro-WEDM. It was found that peak current *I* and pulse on-time duration *t_on_* had the greatest influence on the output quality parameter Ra of the machined surface and the performance parameter of the electrical discharge process when machining HSS with the micro-WEDM technology. In contrast, voltage of discharge *U* had the least influence. Therefore, when designing mathematical regression models, only the parameters peak current *I* and pulse on-time duration *t_on_* were considered. However, as already mentioned above, the productivity of the electrical discharge process, as well as the achieved quality of the machined surface, are defined by a mutual combination of the MTP settings and the properties of the machined material; therefore, in the next step of optimizing the quality of the machined surface and maximizing the productivity of the electrical discharge process in the form of mathematical modeling, these parameters were approached comprehensively.

### 2.3. Regression and Optimization Analysis of the Design

The task of the proposed mathematical regression models was to transform the experimentally obtained data into mathematical notation with the highest possible accuracy [75,76]. Thus, the design of the complex objective function was a key step in optimization of the output-dependent parameters Ra and MRR in relation to the input-independent variables in machining of HSS by micro-WEDM technology [77,78,79]. At the same time, high demands were placed on the accuracy of the mathematical regression models. Their high accuracy can only be achieved if the statistical evaluation of recorded data, regression analysis, and interpretation of the model have been performed correctly. The basic statistical analysis of the proposed mathematical regression model for the prediction of the investigated output-dependent parameter Ra of the quality of the machined surface, and the productivity parameter MRR of the electrical discharge process depending on the change of the investigated independent input variables’ peak current *I*, pulse on-time duration *t_on_*, thermal conductivity *κ*, and specific electric resistance *ρ* was carried out using analysis of variance (ANOVA).

The least squares method (LSM) was applied to build a mathematical regression model for predicting the output quality parameter Ra of the machined area, or the performance parameter MRR in micro-WEDM of HSS, which properly approximated the *n*-tuple of measured values $\left[x_{1},x_{2},\ldots ,x_{m},y\right]$ by the function of *m* variables in the form:

*y = f*(*x*_1_, …, *x_m_*), (1)

where parameter *y* represents the functional dependence of the output quality parameter Ra of the machined surface, or of the performance parameter MRR in micro-WEDM of HSS, while the parameters *x*_1_ to *x_m_* were their real recorded values.

Based on a preliminary analysis of the nature and distribution of the experimentally measured values of the quality parameter of the machined surface Ra, or of the performance parameter MRR in micro-WEDM of HSS, an exponential function based on any natural number in the form (2) was chosen for the mathematical regression model:

(2) $$y=a_{00}\;\cdot \;a_{10}^{x_{1}}\;\cdot \;a_{01}^{x_{2}}\;\cdot \;a_{11}^{x_{1}\cdot x_{2}}.$$

An important condition for establishing the mathematical model was that the function *S*(*A*) expressing the sum of the squares of the differences between the calculated and measured values in all cases reached a minimum according to the equation:

(3) $$S(A)=\sum_{i=1}^{r}{\left[y_{i}-f\left(x_{1},\ldots {,x}_{m},A\right)\right]}^{2}.$$

Subsequently, after the mathematical regression models were built, the selection of a proper optimization method was performed using a suitable software environment [80,81]. The optimization of the response in terms of the qualitative index of the machined surface Ra and the quantitative index MRR of the electroerosion process was based on a suitability analysis. This is a suitable method to find the optimum values in machining of HSS by micro-WEDM technology. The optimization criterion in the given case was the maximization of the productivity of the electrical discharge process given by the parameter MRR while simultaneously achieving a high quality standard of the machined surface through the minimization of the roughness parameter Ra of the machined surface according to the equations:

(4) $$\mathrm{MRR}=\left\{{\left(\frac{x-x_{min}}{x_{max}-x_{min}}\right)}^{vol}\;\left\{\begin{array}{c} 0\to x\leq x_{max}\\ x_{min}\leq x\leq x_{max}\\ 1\to x\leq x_{max} \end{array}\right.\right.,$$

(5) $$\mathrm{Ra}=\left\{{\left(\frac{x-x_{max}}{x_{min}-x_{max}}\right)}^{vol}\;\left\{\begin{array}{c} 0\to x\leq x_{max}\\ x_{min}\leq x\leq x_{max}\\ 1\to x\leq x_{min} \end{array}\right.\right.,$$

where the suitability value of the parameter *x* varied from 0 to 1. In the case of MRR (4), it was required to achieve a suitability value of 1 as its maximum value, and conversely in the case of Ra (5), it was required to achieve a suitability value of 1 as its minimum value. Obtaining a suitability value of 0 was completely undesirable in both cases (4) and (5). At the same time, the requirement of the corresponding response increased with the values of the parameters Ra and MRR. The optimization then could be carried out using the acquired functions, while the potential input parameters of the electrical discharge process when machining HSS were in the case of the physical properties of the machined material thermal conductivity *κ* and specific electric resistance *ρ*, and in the case of MTP peak current *I* and pulse on-time duration *t_on_*.

## 3. Results and Discussion

### 3.1. The Design of Mathematical Models for Minimizing the Roughness Parameter Ra of the Machined Surface during Micro-WEDM of HSS

The next step in the process of optimizing the quality of the machined surface in micro-WEDM of HSS was the design of mathematical regression models in order to minimize the roughness parameter Ra of the machined surface. This consisted of the construction of mathematical regression models describing the relation of the output-dependent qualitative parameter Ra of the machined surface to the combination of settings of important input-independent variables MTP (*I*, *t_on_*) and the physical properties of the machined material (*κ*, *ρ*). To create mathematical models for minimizing the roughness parameter Ra of the machined surface in micro-WEDM of HSS, the method of least squares was applied according to Equation (1), where the parameter *y* represents the functional dependence of the roughness parameter Ra of the machined surface. Parameters *x*_1_ to *x_m_* are its real recorded values.

After adjusting Equations (2) and (3), a set of linear equations was obtained, the solution of which revealed the sought coefficients. Since the qualitative parameter Ra in micro-WEDM of HSS is significantly influenced by *I* and *t_on_*, the mathematical regression model was built based on the approximation of the measured values of the given parameter, as a function of seven variables in the form:

(6) $$\mathrm{Ra}=a_{00}\cdot {a_{10}}^{I}\cdot {a_{20}}^{I^{2}}\cdot {a_{30}}^{I^{3}}\cdot {a_{01}}^{t_{\mathrm{on}}}\cdot {a_{02}}^{{t_{\mathrm{on}}}^{2}}\cdot {a_{03}}^{{t_{\mathrm{on}}}^{3}},$$

which approximates *n*—tuple of measured values (*I*, *t_on_* and Ra) the function dependence

(7) $$\mathrm{Ra}=f\left({I,t}_{\mathrm{on}},A\right)=f\left({I,t}_{\mathrm{on}}{,a}_{00},\ldots {,a}_{\mathrm{ij}}\right),$$

where unknown variables *a_ij_, i, j =* 0, …, *r* are calculated so that the surface *S*(*A*) best approximates the recorded values of the dependent output parameters’ Ra according to the relation:

(8) $$S(A)=\sum_{i=1}^{n}{\left[\mathrm{Ra}-f\left({I,t}_{\mathrm{on}},A\right)\right]}^{2},$$

provided that the given function reaches its minimum. The unknown in this case is the matrix of unknown variables *a_ij_*.

Then, the mathematical regression model describing the dependence of the roughness parameter Ra of the machined surface in micro-WEDM of HSS on the input variable parameters of the process *I* and *t_on_* has the form:

(9) $$\mathrm{Ra}=0.19979\cdot 0.281666^{I}\cdot 1.263728^{I^{2}}\cdot 0.987126^{I^{3}}\cdot 1.594419^{t_{\mathrm{on}}}\cdot 0.983842^{{t_{\mathrm{on}}}^{2}}\cdot 1.000183^{{t_{\mathrm{on}}}^{3}}\;\mathrm{correlation}\mathrm{index}\mathrm{is}{\mathrm{IC}}^{2}=0.9973$$

The accuracy of the established mathematical model is given by the correlation index, which for the quality parameter Ra of the roughness of the machined surface has value of 0.9973, which represents a deviation of the actual measured values from the calculated values of 0.27%.

Analogously, the mathematical regression model was built describing the dependence of the roughness parameter Ra of the machined surface in micro-WEDM of HSS on the process input parameters *κ* and *ρ* with correlation index 0.9511, which corresponds to the deviation of the actual measured values from the calculated values of 4.89%.

(10) $$\mathrm{Ra}=0.000074\cdot 0.000013^{\kappa }\cdot 11821.10461^{\kappa^{2}}\cdot 0.000001^{\kappa^{3}}\cdot 0.00000014^{\rho }\cdot 2.98014^{\rho^{2}}\cdot 0.96492^{\rho^{3}}\;\mathrm{correlation}\mathrm{index}\mathrm{is}{\mathrm{IC}}^{2}=0.9511$$

**Figure 7:**
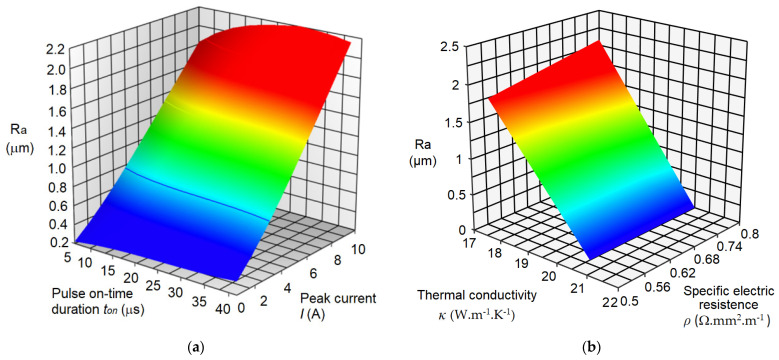
3D graphical dependence of output-dependent variable Ra in micro-WEDM of HSS on input-variable parameters. (**a**) Response of Ra to *I* and *t_on_*; (**b**) response of Ra to *κ* and *ρ*.

Based on the established mathematical regression models (9) and (10), we obtained 3D graphical dependences (Figure 7) of the output quality parameter Ra of the machined surface during micro-WEDM of HSS on the input-variable parameters of the process *I*, *t_on_*, *κ*, and *ρ*.

Several facts can be observed from the graphs in Figure 7, which describe in detail the relations between the output-dependent variable Ra and the input-variable parameters *I*, *t_on_*, *κ*, and *ρ* in micro-WEDM of HSS. First of all, it can be observed that the blue areas in both graphs represent the minimum values of the quality parameter Ra of the roughness of the machined surface, and the red areas, in contrast, its maximum values. Furthermore, it can be observed that the lowest value of the qualitative parameter Ra = 0.24 μm can be achieved at the value of the input MTP of the electrical discharge process *I* = 2A; *t_on_* = 5 μs; *κ* = 21 W·m^−1^·K^−1^; and *ρ* = 0.54 Ω·mm^2^·m^−1^. The maximum value of the roughness parameter of the machined surface Ra = 2.257 μm can be achieved with the values of the input parameters of the electrical discharge process *I* = 8 A; *t_on_* = 40 μs; *κ* = 19 W·m^−1^·K^−1^; and *ρ* = 0.80 Ω·mm^2^·m^−1^.

### 3.2. Design of Mathematical Models for Maximizing the Productivity Parameter MRR in Micro-WEDM of HSS

In the next step of optimizing the quality of the machined surface and the productivity of the electrical discharge process in micro-WEDM of HSS, mathematical regression models were proposed to maximize the MRR parameter. The proposal consisted of the construction of mathematical regression models, describing the relation of the output-dependent performance parameter MRR of the electrical discharge process on the combination of settings of significant input-independent variables MTP (*I*, *t_on_*) and physical properties of the machined material (*κ*, *ρ*). To create mathematical models for maximizing the parameter MRR in micro-WEDM of HSS, the least squares method was applied according to Equation (1), where the parameter *y* represents the functional dependence of the output performance parameter MRR. Parameters *x*_1_ to *x_m_* are its real recorded values.

After adjusting Equations (2) and (3), a set of linear equations was obtained, the solution of which provided the sought coefficients. Since the output performance parameter MRR in micro-WEDM of HSS is significantly influenced by *I* and *t_on_*, the mathematical regression model was built based on the approximation of the measured values of the given parameter as a function of seven variables in the form:

(11) $$\mathrm{MRR}=a_{00}\cdot {a_{10}}^{I}\cdot {a_{20}}^{I^{2}}\cdot {a_{30}}^{I^{3}}\cdot {a_{01}}^{t_{\mathrm{on}}}\cdot {a_{02}}^{{t_{\mathrm{on}}}^{2}}\cdot {a_{03}}^{{t_{\mathrm{on}}}^{3}},$$

which approximates *n*—tuple of measured values (*I*, *t_on_* and MRR) by functional dependence

(12) $$\mathrm{MRR}=f\left({I,t}_{\mathrm{on}},A\right)=f\left({I,t}_{\mathrm{on}}{,a}_{00},\ldots {,a}_{\mathrm{ij}}\right),$$

where unknown variables *a_ij_, i, j =* 0, …, *r* are calculated so that the area *S*(*A*) would best approximate the recorded values of dependent MRR output parameters according to the equation:

(13) $$S(A)=\sum_{i=1}^{n}{\left[\mathrm{MRR}-f\left({I,t}_{\mathrm{on}},A\right)\right]}^{2},$$

provided that the given function reaches its minimum. The unknown in this case is the matrix of unknown variables *a_ij_*.

Then, the mathematical regression model describing the dependence of the output performance parameter MRR in micro-WEDM of HSS on the input-variable process parameters *I* and *t_on_* has the form:

(14) $$\mathrm{MRR}=0.0311\cdot 0.07275^{I}\cdot 1.62571^{I^{2}}\cdot 0.97292^{I^{3}}\cdot 2.01542^{t_{\mathrm{on}}}\cdot 0.9745^{{t_{\mathrm{on}}}^{2}}\cdot 1.0003^{{t_{\mathrm{on}}}^{3}}\;\mathrm{correlation}\mathrm{index}\mathrm{is}{\mathrm{IC}}^{2}=0.9968$$

The accuracy of the established mathematical model is given by the correlation index, which for the performance parameter MRR has the value of 0.9968, which represents deviation of the actual measured values from the calculated values of 0.32%.

Analogously, a mathematical regression model describing the dependence of the output performance parameter MRR in micro-WEDM of HSS on the process input parameters κ and ρ was constructed with a correlation index 0.9258, which corresponds to the deviation of the actual measured values from the calculated values of 7.42%.

(15) $$\mathrm{MRR}=0.000001\cdot 4.74367^{\kappa }\cdot 0.000012^{\kappa^{2}}\cdot 0.16753^{\kappa^{3}}\cdot 0.0000011^{\rho }\cdot 1.92175^{\rho^{2}}\cdot 0.97911^{\rho^{3}}\;\mathrm{correlation}\mathrm{index}\mathrm{is}{\mathrm{IC}}^{2}=0.9258$$

**Figure 8:**
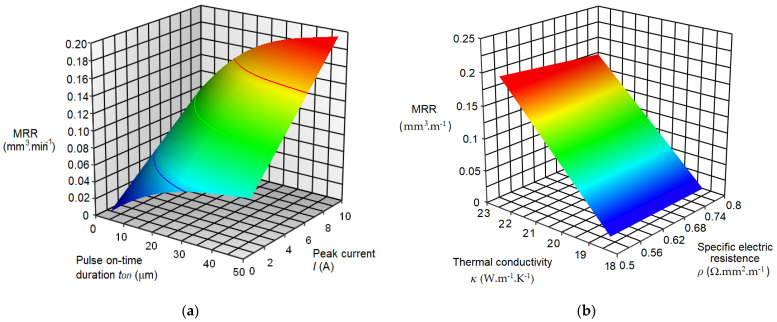
3D graphical dependence of the output-dependent variable MRR in micro-WEDM of HSS on the input variable parameters. (**a**) Response of MRR to *I* and *t_on_*; (**b**) response of MRR to *κ* and *ρ*.

Based on the established mathematical regression models (14) and (15) with the application of the simulation program, 3D graphical dependences (Figure 8) of the output performance parameter MRR of the electrical discharge process in micro-WEDM of HSS on the input variable process parameters *I*, *t_on_*, *κ*, and *ρ* were created.

The graphs in Figure 8 show the relation between the output-dependent variable MRR and the input-variable parameters *I*, *t_on_*, *κ*, and *ρ* in micro-WEDM of HSS. It can be observed that the red area in both graphs represents the maximum value of the output power parameter MRR of the electrical discharge process, and the blue area, in contrast, its minimum value. Furthermore, it can be observed that the highest value of the output power parameter MRR = 0.204 mm^3^·min^−1^ was achieved with the input MTP values of electrical discharge process *I* = 8 A; *t_on_* = 40 μs; *κ* = 21 W·m^−1^·K^−1^; and *ρ* = 0.54 Ω·mm^2^·m^−1^. The minimum value of the output performance parameter MRR = 0.014 mm^3^·min^−1^ was achieved with the input values of the electrical discharge process parameters *I* = 2A; *t_on_* = 5 μs; *κ* = 19 W·m^−1^·K^−1^; and *ρ* = 0.80 Ω·mm^2^·m^−1^.

### 3.3. Optimization of the Output Qualitative Parameter Ra of the Machined Surface with Regard to Maximizing the Performence of the Electrical Discharge Process in Micro-WEDM of HSS

Based on the obtained mathematical regression models (9), (10), (14), and (15) for the prediction of the output qualitative parameter Ra of the machined surface, and the output performance parameter MRR of the electrical discharge process in micro-WEDM of HSS depending on the input variable process parameters *I*, *t_on_*, *κ*, and *ρ*, the 2D graphical optimization of the output parameters was performed.

**Figure 9:**
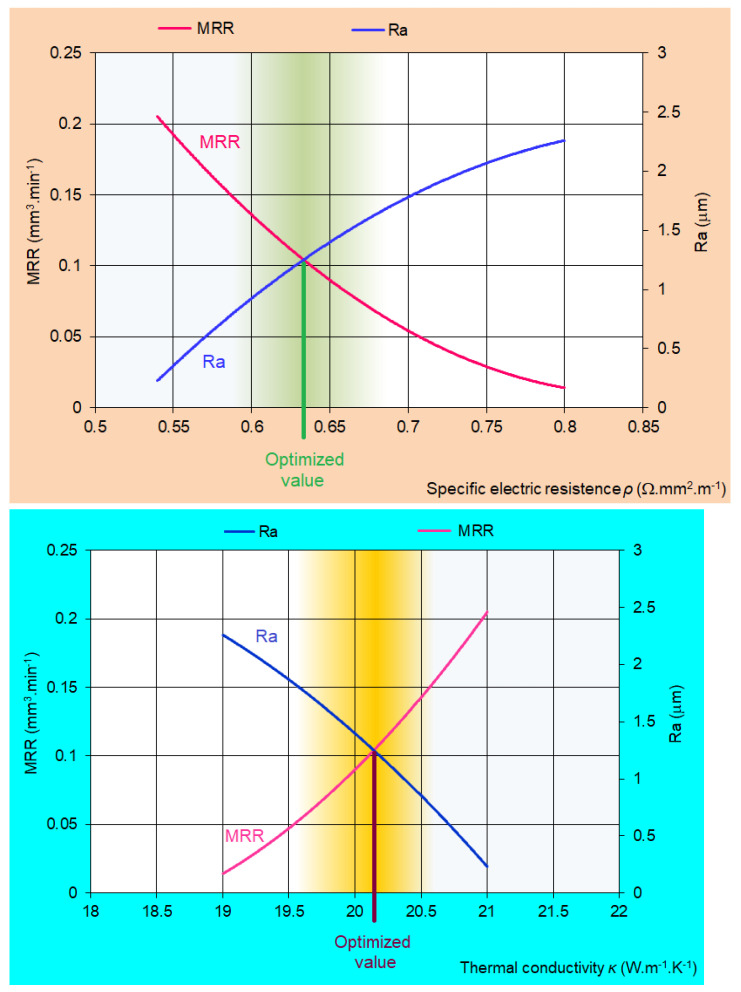
Optimization of output-dependent parameters Ra and MRR in micro-WEDM of HSS in relation to input-variable parameters *κ* a *ρ*.

Based on the performed analysis of the proposed regression models (10) and (15), several facts can be stated. It can be concluded that with the increasing value of the thermal conductivity parameter *κ* and the decreasing value of the specific electric resistance parameter *ρ* in micro-WEDM tool steels, there was an increase of the value of the performance parameter MRR of the electrical discharge process to the level of 0.204 mm^3^·min^−1^. However, on the other hand, there was a decrease in the overall quality of the machined surface in terms of the roughness parameter Ra of the machined surface. In this case, its value was at the level of 2.257 μm. The best quality of the machined surface in terms of the roughness parameter of the machined surface Ra = 0.24 μm was achieved with high values of the input parameter *κ* and low values of the parameter *ρ*. However, at these low values of the input parameter settings, there was a significant decrease of the output parameter MRR of productivity of the electrical discharge process to the level of 0.014 mm^3^·min^−1^. For this reason, it was necessary to perform their mutual optimization. Graphical dependencies in Figure 9 describe the mutual optimization of the output quality parameter Ra of the machined area and the output performance parameter MRR of the productivity of the electrical discharge process in micro-WEDM of HSS in relation to the significant input-variable parameters *κ* and *ρ*.

From the graphic optimization in Figure 9, it can be concluded that in micro-WEDM of HSS, the optimal value of the output quality parameter of the machined surface was Ra = 1.2 μm and the output performance parameter of the productivity of the electrical discharge process MRR = 0.10 mm^3^·min^−1^. These optimal values of output-dependent parameters of the process were achieved at the value of input parameters *κ* = 20.1 W·m^−1^·K^−1^ and *ρ* = 0.64 Ω·mm^2^·m^−1^.

**Figure 10:**
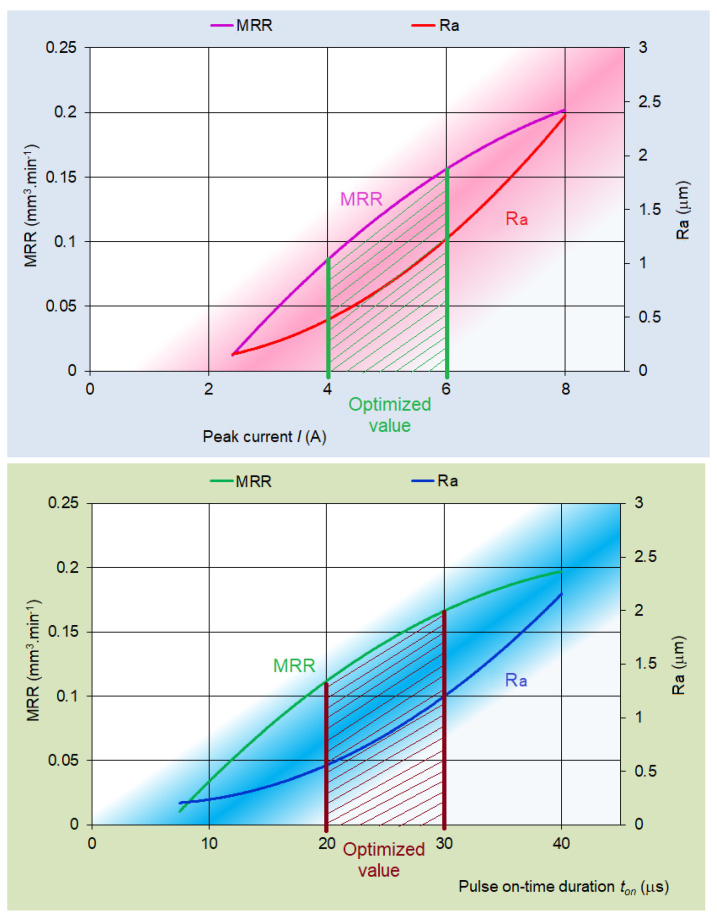
Optimization of output-dependent parameters Ra and MRR in micro-WEDM of HSS in relation to input-variable parameters *I* a *t_on_*.

Based on the analysis of the proposed mathematical regression models (9) and (14), several facts can be stated. It can be concluded that with the increasing value of the peak current *I* and the pulse on-time duration *t_on_* during micro-WEDM of tool steels, there was an increase of the performance parameter of the electrical discharge process MRR = 0.204 mm^3^·min^−1^. On the other hand, however, there was a decrease of the overall quality of the machined surface in terms of the surface roughness parameter. In this case, its value was at the level of Ra = 2.257 μm. The highest quality of the machined surface in terms of the surface roughness parameter Ra = 0.24 μm was observed at low values of process MTP inputs *I* = 2 A and *t_on_* = 5 μs. However, at these values of the input MTP settings, there was a significant drop of the output parameter of the productivity of the electrical discharge process to the level of MRR = 0.014 mm^3^·min^−1^. For this reason, it was necessary to perform their mutual optimization. Graphical dependences in Figure 10 describe the mutual optimization of the output quality parameter Ra of the machined area and the output performance parameter MRR of the productivity of the electrical discharge process in micro-WEDM of HSS in relation to the significant input-variable parameters of the process *I* a *t_on_*.

From the shown graphic optimization in Figure 10, it can be concluded that in micro-WEDM of HSS, the optimal value of the output quality parameter Ra of the machined surface was in the range of 0.5 to 1.2 μm and the output performance parameter MRR of the productivity of the electrical discharge process was in the range of 0.085 to 0.17 mm^3^·min^−1^. These optimal values of output-dependent parameters of the process were achieved by setting peak current *I* in the range of 4.0 to 6.0A and pulse on-time duration *t_on_* in the range of 20.0 to 30.0 μs.

The experimental research was carried out according to DoE considering four input technological parameters (*I*, *t_off_*, *t_on_*, *U*) of the electrical discharge process and at the same time selected four physical (*ρ*, *κ*) and mechanical (*κ*, HRC) indicators of the properties of the processed materials. To support the optimization of Ra and MRR parameters, regression mathematical models were proposed as a function of seven variables using the least squares method (LSM). It was found that all considered process input parameters did not have the same effect on Ra and MRR responses in micro-WEDM of HSS. Therefore, optimization was applied with the inclusion of significant output factors of the electrical discharge process. The best results for the response Ra = 0.24 μm shared the parameters *I* = 2 A, *t_on_* = 5 μs, *ρ* = 0.80 Ω·mm^2^·m^−1^, and *κ* = 19 W·m^−1^·K^−1^, and for the response MRR = 0.204 mm^3^·min^−1^ parameters *I* = 8 A, *t_on_* = 40 μs, *ρ* = 0.54 Ω·mm^2^·m^−1^, and *κ* = 21 W·m^−1^·K^−1^. Within the performed experimental research, it was found that of the four considered input MTP variables of the electrical discharge process *I*, *t_on_*, *t_off_*, and *U*, the peak current *I* and pulse on-time duration *t_on_* had the greatest influence on the output quality parameter Ra of the machined surface roughness and the output performance parameter MRR in micro-WEDM of HSS. It was found that with the increasing values of the input MTP variables of the electrical discharge process *I* and *t_on_*, there was a significant increase of the output performance parameter MRR of the electrical discharge process and a deterioration of the output quality parameter Ra of the machined surface roughness. At the same time, the highest value of output-dependent parameters MRR = 0.204 mm^3^·min^−1^ and Ra = 2.257 μm was achieved with the combination of process MTP input values of *I* = 8.0 A and *t_on_* = 40 μs. Conversely, the lowest values of MRR = 0.014 mm^3^·min^−1^ and Ra = 0.24 μm were achieved with the combination of process MTP input values of *I* = 2.0 A and *t_on_* = 5 μs. The dependence of the values of the output parameters Ra and MRR during micro-WEDM of HSS on the value of the input parameters *κ* and * ρ* was recorded. At the same time, the highest value of output-dependent parameter MRR = 0.204 mm^3^·min^−1^ and the lowest value of output-dependent parameter Ra = 0.24 μm was recorded with the combination of input parameters *κ* = 21 W·m^−1^·K^−1^ and *ρ* = 0.54 Ω·mm^2^·m^−1^. In contrast, the lowest value of the output-dependent parameter MRR = 0.014 mm^3^·min^−1^ and the highest value of the output-dependent parameter Ra = 2.257 μm was recorded with the combination of input parameters *κ* = 19 W·m^−1^·K^- 1^ and *ρ* = 0.80 Ω·mm^2^·m^−1^. By the means of the proposed mathematical regression models with subsequent simulation, a local maximum of 0.204 mm^3^·min^−1^ of the productivity parameter MRR of the electrical discharge process was recorded, and at the same time a local minimum of 0.24 µm of the quality parameter Ra of the machined surface was recorded.

## 4. Conclusions

The experimental research carried out was aimed at eliminating the shortcoming associated with low performance in machining of HSS by micro-WEDM technology and at the same time achieving a high quality standard of the machined surface in terms of the surface roughness parameter Ra. The solution was designed by optimizing the output-dependent quality indicator of the machined surface Ra and the output performance parameter MRR of the electroerosion process. The optimization of these parameters was performed in relation to the significant input parameters related to the physical properties (*ρ*, *κ*) of the machined HSS, and additionally, in relation to the significant input-independent variables MTP of the electroerosion process (*I*, *t_o__n_*). At the same time, the optimization was performed with regard to maximizing the output performance parameter MRR of the electroerosion process while maintaining a high quality standard of the machined surface in terms of the parameter Ra. Mathematical regression models were obtained, which allowed the simulation and prediction of the output-dependent quality indicator of the machined surface Ra and also the output performance indicator MRR of the electroerosion process in the machining of HSS by micro-WEDM technology. In the first step, based on the obtained mathematical regression models, the optimization of the parameters Ra and MRR in relation to the properties (*ρ*, *κ*) of the machined HSS was carried out. The second step was the identification of a proper range of significant MTP input variables (*I*, *t_o__n_*). By the performed optimization with the orientation to maximize the process performance parameter MRR in the range of 0.085 to 0.17 mm^3^·min^−1^ and a favorable value of the quality parameter of the machined area Ra in the range of 0.5 to 1.2 μm in machining of HSS by micro-WEDM technology, for *κ* with a value of 20.1 W·m^−1^·K^−1^ and *ρ* with a value of 0.64 Ω·mm^2^·m^−1^, values of significant output-independent MTP peak current in the range of 4.0 to 6.0 A and pulse on-time duration in the range of 20.0 to 30.0 μs were identified.

## Figures and Tables

**Table 1:** Basic mechanical and physical properties of materials of experimental samples.

Properties of Steel	HSS		
	EN HS 3-3-2	EN HS 10-5-3-10	EN HS6-5-2C
Specific electric resistence *ρ* (Ω·mm^2^·m^−1^)	0.65	0.8	0.54
Thermal conductivity at 20 °C *κ* (W·m^−1^·K^−1^)	20	19	21
Tensile strength Rm (MPa) (in natural state)	757	790	1158
Achievable hardness after refining HRC	65	67	62

**Table 2:** Chemical composition of HSS used for the production of experimental samples.

Marking of Steel	Chemical Composition in %							
	C	Mn	Si	Cr	Mo	V	W	Co
EN HS 3-2-2	0.95–1.03	max.0.45	max.0.45	3.8–4.5	2.5–2.8	2.2–2.5	2.7–3	-
EN HS 10-5-3-10	1.15–1.30	max.0.45	max.0.45	3.8–4.6	3.5–4.3	3.0–3.7	9.5–11.0	10.0–11.5
EN HS6-5-2C	0.86–0.94	max.0.40	max.0.45	3.80–4.50	4.70–5.20	1.70–2.10	5.90–6.70	-

**Table 3:** The range of MTP settings applied in the production of experimental samples from HSS by micro-WEDM technology.

MTP	Setting Level	Parameter Setting Value	The Expected Value of the Parameter	
			MRR	Ra
Peak current *I* (A)	high	8.0	high	high
	middle	6.0		
	low	2.0	low	low
Pulse on-time duration *t_on_* (μs)	high	40.0	high	high
	middle	20.0		
	low	5.0	low	low
Pulse off-time duration *t_off_* (μs)	high	15.0	low	low
	middle	9.0		
	low	3.0	high	high
Voltage of discharge *U* (V)	high	90	low	low
	middle	85		
	low	70	high	high

**Table 4:** Basic parameters of CHMER EDM G32F.

Basic Technical Parameters of Electrical Discharge Machine CHMER EDM G32F	
Portal X/Y/Z	360 × 250 × 220 mm
Workpiece size X/Y/Z	725 × 560 × 215 mm
Workpiece weight	300 kg
Wire diameter range	0.015–0.3
Wire feed rate	300 mm/s
Wire tension	300–2500 gf

## Data Availability

Data are contained within the article.

## Nomenclature

HRC	Rockwell hardness
HSS	high speed steels
IC	index correlation
MRR	material removal rate
Ra	parameter of surface roughness (µm)
*S*(*A*)	function expressing the sum of squared differences
*I*	peak current (A)
*t_on_*	pulse on-time duration (µs)
*t_off_*	pulse off-time duration (µs)
*U*	voltage of discharge (V)
*y*	desired value function
*x*	measured values
*x*_*min/max*_	lower/upper response limit values
Micro-WEDM	micro-wire electrical discharge machining
